# Bisulfite Pretreatment Improves Enzymatic Digestibility of Oil Palm Empty Fruit Bunch and Poplar Through Changing Its Structure and Lignin Distribution

**DOI:** 10.3390/ijms26115334

**Published:** 2025-06-01

**Authors:** Liping Tan, Xuezhi Li, Xianqin Lu, Jian Zhao

**Affiliations:** 1 State Key Laboratory of Microbial Technology, Shandong University, Qingdao 266237, China; tanliping163@163.com (L.T.); lixz@sdu.edu.cn (X.L.); 202299900076@sdu.edu.cn (X.L.); 2 Shandong Provincial Key Laboratory of Microbial Engineering, Qilu University of Technology (Shandong Academy of Sciences), Jinan 250353, China

**Keywords:** empty fruit bunch (EFB), poplar, bisulfite pretreatment, ultrastructure, lignin distribution

## Abstract

This paper investigated the changes in anatomy, ultrastructure and lignin distribution of oil palm empty fruit bunch (EFB) by bisulfite pretreatment. It was found that after bisulfite pretreatment, a large number of pores formed in the cell walls, and the removal of part of the lignin in the cell wall corner, partial middle layer, and other locations made the tissue structure of the EFB looser, which uncovered cellulose and broke the steric hindrance of cellulase access to cellulose in EFB, and also weakened the negative influence of lignin on cellulase. The changes can greatly contribute to the improvement of enzymatic hydrolysis after bisulfite pretreatment, which is consistent with the increased saccharification efficiency of the pretreated EFB. Poplar was also used to compare the differences and similarities between non-wood and wood materials.

## 1. Introduction

As a major byproduct of the palm oil industry, empty fruit bunch (EFB) has been recognized as one of the most potential material sources for bioethanol production in Southeast Asia, especially in Malaysia and Indonesia [1]. Unfortunately, the high natural recalcitrance for enzymatic hydrolysis remains a major challenge for the bioethanol production [2]. To minimize the recalcitrance and increase enzymatic digestibility of EFB, effects of various pretreatment processes on the enzymatic digestibility of EFB have been assessed, including dilute maleic acid pretreatment [3], steam pretreatment [4], hydrothermal and oxalic acid pretreatment [5], combined with acid-alkaline pretreatment [6], organosolv pretreatment [7], ammonia fiber explosion (AFEX) combined with NaOH pretreatment [8], bisulfite pretreatment [9], ultrasonication with deep eutectic solvent (DES) pretreatment [10], and so on. For example, in our previous work, the effect of bisulfite pretreatment was investigated, and proved that it is an effective and practical method for enhancing enzymatic hydrolysis of EFB for bioethanol production [9]. The conversion of cellulose to glucose can reach 81% after 72 h of enzymatic hydrolysis for bisulfite-pretreated EFB, which is increased by 322% compared with untreated EFB (19.2% of cellulose conversion). An ethanol concentration of 52 g/L in fermentation liquor could be obtained by quasi-simultaneous saccharification and fermentation (Q-SSF) process using the bisulfite-pretreated EFB.

To interpret the reason for improved enzymatic hydrolysis by bisulfite pretreatment, some changes in the EFB materials before and after bisulfite pretreatment have also been investigated, such as chemical components of EFB, crystallinity of cellulose, chemical structure and properties of hemicellulose and lignin, specific area of EFB, and adsorption characteristics of cellulase onto lignin [11]. It was shown that bisulfite pretreatment can partially hydrolyze the amorphous hemicellulose in hot acid solution reaction, leading to relatively increased cellulose crystallinity. Meanwhile, EFB materials were separated into individual fibers, as well as the bigger pore sizes, resulting in increased specific area of EFB during bisulfite pretreatment. In addition, the molecular weights of hemicellulose fractions that are isolated from the bisulfite-pretreated EFB are decreased compared with that from untreated EFB because of the degradation of high molecular-weight hemicellulose during bisulfite pretreatment. In addition, partial removal of methoxyl groups and syringyl units, formation of methyl and methylene groups, and esterification of aromatic and alcohol groups of the propane chain in lignin fractions occur during bisulfite pretreatment. All the changes such as degradation and removal of hemicellulose, increased specific area, changed functional groups, etc., led to the increased accessibility of enzyme to cellulose, and decreased nonproductive adsorption of enzyme onto lignin, revealing the underlying mechanism of improved enzymatic hydrolysis of EFB by bisulfite pretreatment to a certain extent.

The natural recalcitrance of lignocellulose originates not only from the chemical compositions of the cell wall (cellulose, hemicellulose, and lignin), but also from the details of their microstructure and distribution regulation at micron scales [12]. Generally speaking, chemical complexity and compact organization of cell walls make the plants extremely recalcitrant. The architecture of cell walls together with chemical characterization would also provide insights into the mechanisms that contribute to the inherent recalcitrance of cell walls [13]. Shamsudin et al. [14] have investigated the morphological alterations of four different types of EFB surfaces, horizontal cross sections and longitudinal sections before and after steam autohydrolysis pretreatment using scanning electron microscopy (SEM). As a result, the rigid and solid materials on the surface of EFB seem to be removed by the pressure and heat from steam pretreatment, leading to smooth and clear surface. The horizontal cross sections showed that the existence of pores in the pretreated EFB may facilitate the penetration of cellulase, thus improving the enzymatic hydrolysis. A destroyed cell wall of pitted vessels is observed from the longitudinal view of steam pretreated EFB. Moreover, the compact and intact surface of EFB change to the loose and irregular structure, suggesting more separated-soften fiber bundles after microwave pretreatment [15]. A large amount of fractures surface EFB fibers that were pretreated by microwave pretreatment can greatly increase the reactive surface area and improve the enzymatic hydrolysis. In our previous study, the morphological changes caused by bisulfite pretreatment were also simply observed using SEM and found that bisulfite pretreatment can separate EFB materials into individual fibers [11]. The fiber separation and fibrillation led to a loosened fiber cell wall with significant delamination. Cell wall structures include anatomical structure and ultrastructure; however, to data, existing studies predominantly employed SEM to observe the structural and surface morphological changes in EFB before and after different chemical pretreatments. For instance, it was observed that EFB subjected to liquid hot water (LHW) pretreatment exhibited pore or crack formation on its surface [16], while acid-treated EFB showed significant surface structural damage [17]. Only few studies about changes in the ultrastructure of EFB cell wall during pretreatment have been reported, especially on bisulfite pretreated EFB. Investigating the changes in nanoscale architecture of EFB cell walls before and after pretreatment can deepen the understanding of the fundamental mechanisms responsible for the inherent recalcitrance of cell walls.

In the present study, the changes in architectural structure of EFB such as anatomical structure of EFB and ultrastructure of cell wall during bisulfite pretreatment were studied by multiple microscopic techniques, including light microscopy (LM), SEM, and atomic force microscopy (AFM). Using chemical analysis and confocal laser scanning microscopy (CLSM), respectively, the changes in the chemical components of EFB and lignin distribution in the cell wall of EFB before and after bisulfite pretreatment were also studied. At the same time, poplar was also used in this study for comparing the differences and similarities between EFB (non-wood) and wood materials. The current work would help us further understand the mechanism of bisulfite pretreatment in improving enzymatic hydrolysis from another point of view.

## 2. Results and Discussion

### 2.1. Improved Enzymatic Digestibility of EFB and Poplar by Bisulfite Pretreatment

Table 1 shows the yields of glucose obtained by enzymatic hydrolysis of EFB and poplar before and after bisulfite pretreatment. It was shown that the glucose yields of untreated EFB and poplar after 72 h of enzyme hydrolysis were only 0.078 g/g EFB and 0.088 g/g poplar, respectively. After bisulfite pretreatment, the glucose yields were 0.378 g/g EFB and 0.312 g/g poplar, respectively, indicating that bisulfite pretreatment effectively enhanced enzymatic hydrolysis of EFB and poplar.

### 2.2. Changes in Chemical Compositions of EFB and Poplar by Bisulfite Pretreatment

To elucidate the reasons for the improvement of enzymatic hydrolysis of two raw materials after bisulfite pretreatment, firstly, their chemical compositions changes were investigated. Table 1 presents the changes in chemical compositions of EFB and poplar after bisulfite pretreatment. As can be seen in Table 1, the ethanol-extractives contents in the pretreated EFB and poplar (7.6% and 10.6%, respectively) greatly increased in comparison with untreated samples (1.5% and 1.3%, respectively). Acidification appeared to be the main reason for the precipitation of extractives on the substance surfaces [18]. Lignin derivatives might precipitate from the pretreatment liquor and be adsorbed by the substance surface in acid condition [19], which led to increased extractives in the bisulfite-pretreated samples. 

It was found that, before pretreatment, the EFB contained more xylan (23.1% and 14.6%, respectively) and less glucan (34.7% and 43.7%, respectively) than poplar, while they had the similar content of lignin. After bisulfite pretreatment, most of the xylan in EFB (79.4%) and poplar (87.3%) were apparently dissolved, whereas such pretreatment removed 39.0% and 33.8% of lignin from untreated EFB and poplar, respectively, indicating that more lignin and relatively less xylan were removed from EFB than that from poplar under the same pretreatment conditions. This phenomenon may be explained by previous studies [11,20] that proved hemicelluloses can be removed through the cleavage of glycosidic ether linkages between sugar units during bisulfite pretreatment, while the removal of lignin is possibly attributed to sulfonation of lignin during bisulfite pretreatment, leading to the formation of lignosulfonate. Under acidic pretreatment conditions used in this study, hemicelluloses such as xylan, due to its lower degree of polymerization and branched structure, are easily subjected to acidic degradation to produce soluble sugars, resulting in a large amount of xylan in EFB and poplar being dissolved out. It was reported that the hemicellulose in non-wood lignocellulosic raw materials contains a higher content of acetyl groups [21]. These acetyl groups are easily peeled during bisulfite pretreatment to form acetic acid, which further intensifies the acidic hydrolysis of hemicellulose. Although a larger amount of xylan was removed from EFB compared to poplar, the higher content of xylan in EFB resulted in a relatively lower calculated removal percentage. The removal of hemicellulose disrupts the cell wall structure, leading to increased porosity. This facilitates the penetration of chemicals into the cell wall to react with lignin, resulting in more lignin being removed from EFB.

After bisulfite pretreatment, compared with the pretreated poplar, the pretreated EFB had similar lignin content (20.5% vs. 21.3%) and relatively high xylan content (7.2% vs. 2.6%). A previous study reported that xylan removal was the dominant factor affecting cellulose saccharification [22]. The relatively high xylan content under the condition of almost the same lignin content is the least beneficial to the enzymatic hydrolysis of cellulose in lignocellulose substrate because of its physical hindrance on cellulose; the conversion of cellulose to glucose for the pretreated EFB was higher than that for the pretreated poplar (0.378 g/g and 0.312 g/g, respectively) in the study possibly due to the different microstructure and distribution of chemical components in the cell wall. The recalcitrance of biomass is a structural and chemical property for inhibiting enzymatic hydrolysis on cell walls, which originates not only from the chemical compositions of the cell wall polymers (cellulose, hemicelluloses, and lignin) but also from the details of their microstructure and distribute regulation at micron scales [12]. Table 1 also reveals that only a small portion of cellulose was lost from EFB and poplar after bisulfite pretreatment (only a loss of 1.8% and 4.0%, respectively).

### 2.3. Changes in Anatomical and Ultrastructure Features of EFB and Poplar Cell Walls by Bisulfite Pretreatment

#### 2.3.1. Changes in Anatomical Structure

**Figure 1:**
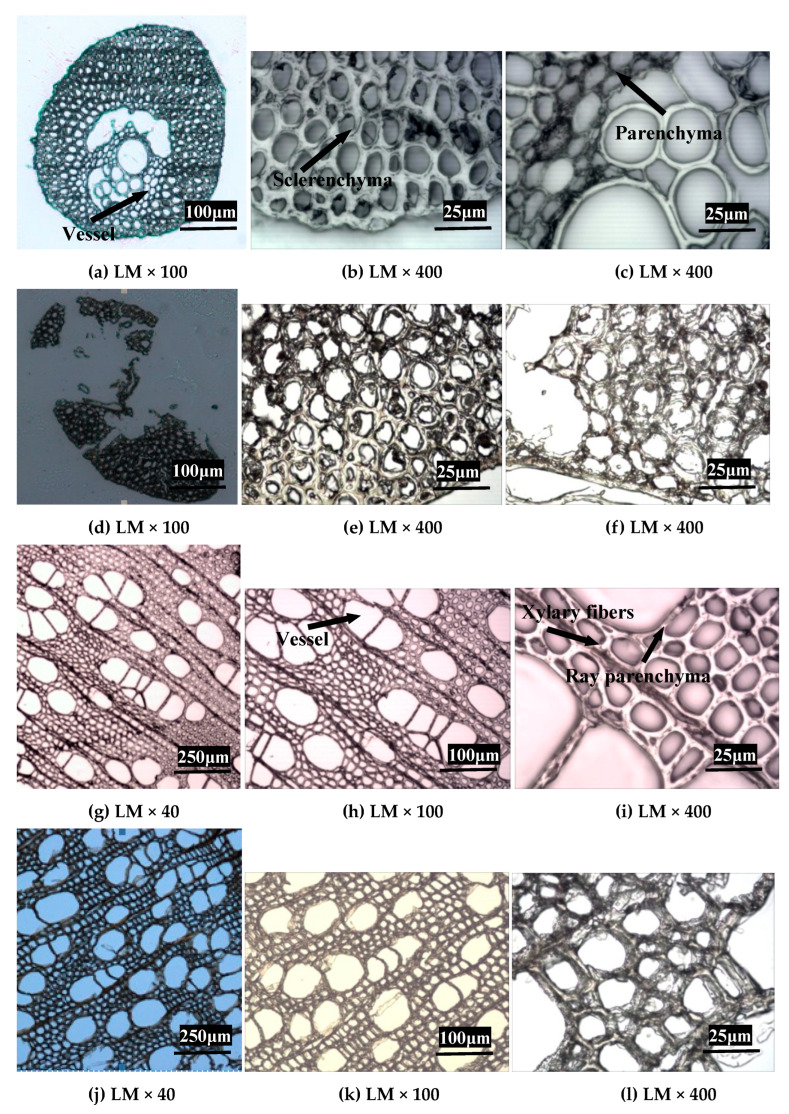
Light microscope (LM) observations of EFB and poplar before and after bisulfite pretreatment. In which, (**a**–**c**): untreated EFB; (**d**–**f**): bisulfite pretreated EFB; (**g**–**i**): untreated poplar; (**j**–**l**): bisulfite pretreated poplar; in which, (**b**,**e**): sclerenchyma of EFB; (**c**,**f**): parenchyma of EFB.

**Figure 2:**
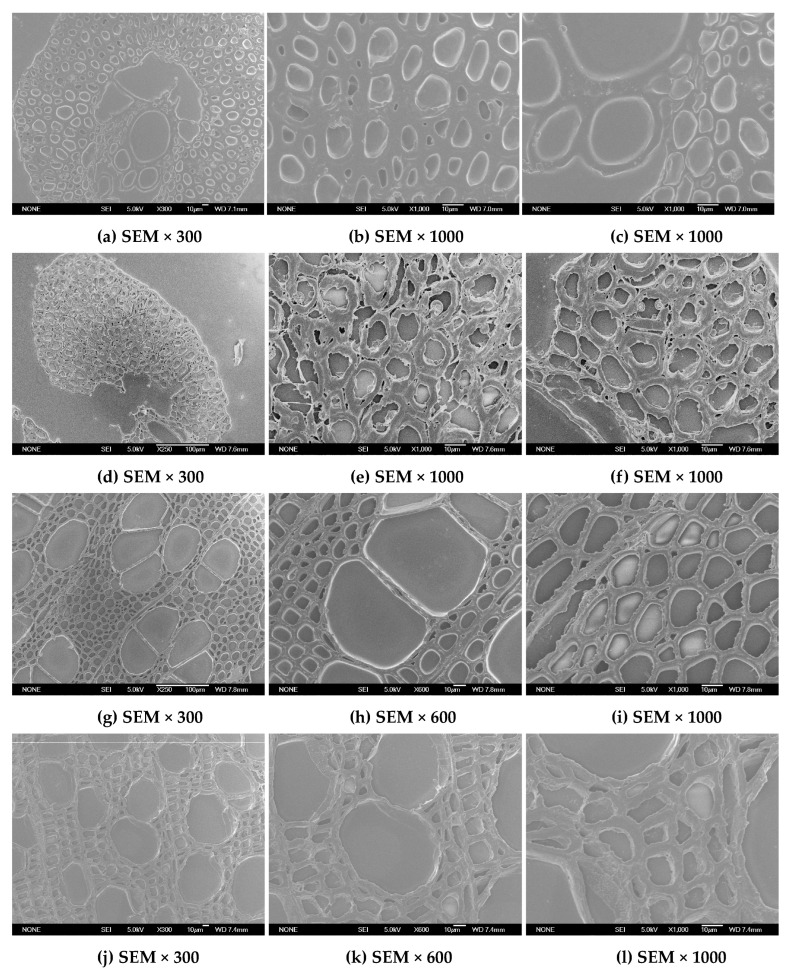
SEM observations of EFB and poplar before and after bisulfite pretreatment. In which, (**a**–**c**): untreated EFB; (**d**–**f**): bisulfite pretreated EFB; (**g**–**i**): untreated poplar; (**j**–**l**): bisulfite pretreated poplar; in which, (**b**,**e**): sclerenchyma of EFB; (**c**,**f**): parenchyma of EFB.

Changes in anatomical features of EFB and poplar were investigated using LM and SEM. It showed that the untreated EFB mostly consisted of parenchyma cells, sclerenchyma cells, vessels, and epidermal cells (Figure 1a and Figure 2a). A large quantity of sclerenchyma cells with thicker and smaller lumen formed the raw material of EFB physical support. The vessels were centrally located in the middle part, and the parenchyma cells were surrounded by the vessels which were connected with the sclerenchyma cells and vessels. For untreated EFB, the length and width of the fiber cell were 0.674 mm (Lw) and 17.234 µm, respectively, and the fines content was 24.35% (Table 2).

After bisulfite pretreatment, the overall tissue structure of EFB was destroyed (Figure 1d–f and Figure 2d–f). Due to sulfite liquor penetration during pretreatment and reaction inside the cell wall, sclerenchyma cells were separated, resulting in destroyed or even broken cell walls. The vessels and parenchyma cells located in the middle part were also damaged during bisulfite pretreatment. The fiber was shortened, the width was decreased, and the fines content was increased after bisulfite pretreatment (Table 2).

Figure 1g–i and Figure 2g–i indicated that the poplar was diffuse-porous wood without distinct growth ring boundaries in the transverse section. The vessels were grounded in the fibrous tissue, which has been reported in many literatures [23,24]. Poplar consists of xylary fibers, vessels, and typically rectangular ray parenchyma. The relatively larger vessel can be responsible for the transport of water and inorganic salt in plants. The wood ray cells in the poplar are exclusively composed of ray parenchyma cells. Xylary fibers located between vessels and ray parenchyma comprise relatively larger proportions of the total cell. After bisulfite pretreatment, the framework of poplar was not damaged, but the structure of cell walls which had tight junction of the untreated poplar became loose (Figure 1j–l and Figure 2j–l). Parts of the cell walls were broken or even fragmented, which could be attributed to sulfite liquor penetration during pretreatment process and reaction inside the cell wall, resulting in the partial removal of hemicellulose and lignin.

The changes in anatomical structures of biomass materials before and after bisulfite pretreatment could give a visual explanation for why the enzymatic digestibility of EFB and poplar are improved by bisulfite pretreatment. It was conceivable that a large number of pores formed on the cell walls in EFB and poplar because of the partial removal of hemicelluloses and lignin during bisulfite pretreatment. Our previous study [11] showed that the specific surface area of the EFB by BET method was increased (from 0.99 to 1.18) after bisulfite pretreatment. Moreover, the volume of pores with diameters of 50 nm or higher was also increased after bisulfite pretreatment, which was beneficial to the penetration of enzymes into the substrate and the release of sugar resulted from enzymatic hydrolysis. All the factors enhanced enzymatic hydrolysis of cellulose. Previous studies have reported that the lignin was the inhibitory biopolymer for the enzymatic hydrolysis of lignocellulosic substrates [25,26,27]. Therefore, partial removal of lignin during bisulfite pretreatment could decrease the negative effect of lignin on enzymatic hydrolysis of cellulose, leading to improved enzymatic efficiency of cellulase.

#### 2.3.2. Changes in Ultrastructure

**Figure 3:**
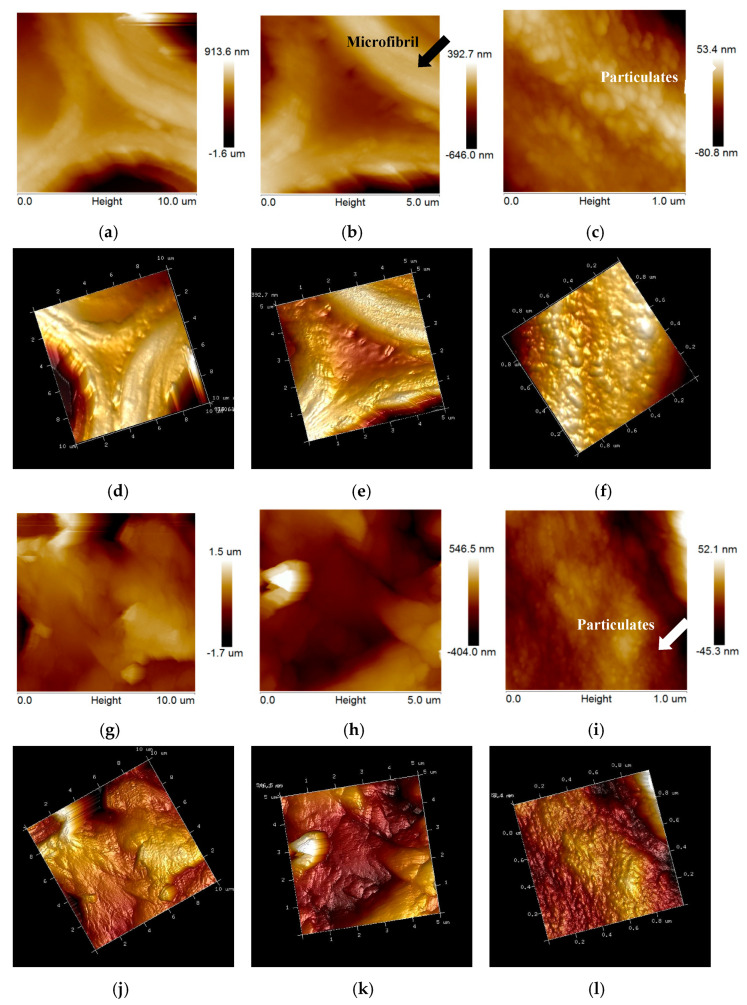
AFM observations of untreated EFB and bisulfite pretreated EFB. In which, (**a**–**c**): phase images of untreated EFB; (**d**–**f**): three-dimensional images of untreated EFB; (**g**–**i**): phase images of bisulfite pretreated EFB; (**j**–**l**): three-dimensional images of bisulfite pretreated EFB.

AFM has been applied to the structural studies of plant cell wall, pretreated substrates and the main components, such as cellulose, lignin, and extractives, for several years. AFM can be used to obtain accurate images of cellulose, plant cell wall surface, and precipitation of lignin on cellulose surface and so on under physiological conditions with nanometer resolution [28,29]. However, many published studies have only focused on changes in the surface ultrastructure of the raw materials and pretreated samples from different pretreatment processes. For example, AFM can be used to measure the ultrastructure of the sample surface of both native and thermal chemically pretreated forms [30]. In the present study, the internal ultrastructure of EFB and bisulfite pretreated EFB was observed by AFM using transverse sections of samples (Figure 3). Phase images and three-dimensional images (Figure 3a–f) showed that the scanning area was located around the cell corner, and the strongly parallel orientation of the microfibrils with characteristic microfibril orientation angles smaller than ±30° might belong to the S2 cell wall region [31]. Besides the cellulose microfibrils, there were also noncellulosic components, such as hemicelluloses, lignin, and extractives (e.g., waxes and other lipophilic extractives), in the cell wall. Figure 3c,f show that particulate matters were distributed in the cell wall of untreated EFB, which might be lignin and extractives [32]. Figure 3a–f show that the surface of the untreated EFB transverse section appeared to be rough because the fiber was combined with a large number of lignin and extractives. Such an appearance verifies the theory that cellulose, hemicellulose, and lignin were mixed with each other in the cell wall. By comparing images of untreated EFB (Figure 3a–f) and pretreated EFB (Figure 3g–l), it clearly shows that bisulfite pretreatment changed the ultrastructure of EFB. After pretreatment, the structure of EFB became rugged with the scanning area of 10 µm × 10 µm (Figure 3a for untreated EFB vs. Figure 3g for treated EFB), the shapes of particulate matters in cell wall of EFB became smaller (Figure 3c for untreated EFB vs. Figure 3i for treated EFB), and the structure of EFB became locally highly smooth with the scanning area of 1.0 µm × 1.0 µm. Particulate matter formed spoke for an amorphous material rather than for a fibrillar crystalline matter, suggesting that the dark particulates were hemicellulose rather than cellulose [31]. A much smaller granular surface after bisulfite pretreatment might be caused by the partial removal of the lignin and extractives in the cell wall of EFB during bisulfite pretreatment. On the other hand, substances with small molecular weight formed due to the cracking of macromolecular substances (for example, hemicellulose, lignin, etc.) [32]. The relative smooth surface after bisulfite pretreatment should be favorable to enzymatic hydrolysis by cellulase, while the irregular and rugged surface before pretreatment reduced cellulase activity, therefore leading to the hydrolysis slowing down as reported in the literature [33].

**Figure 4:**
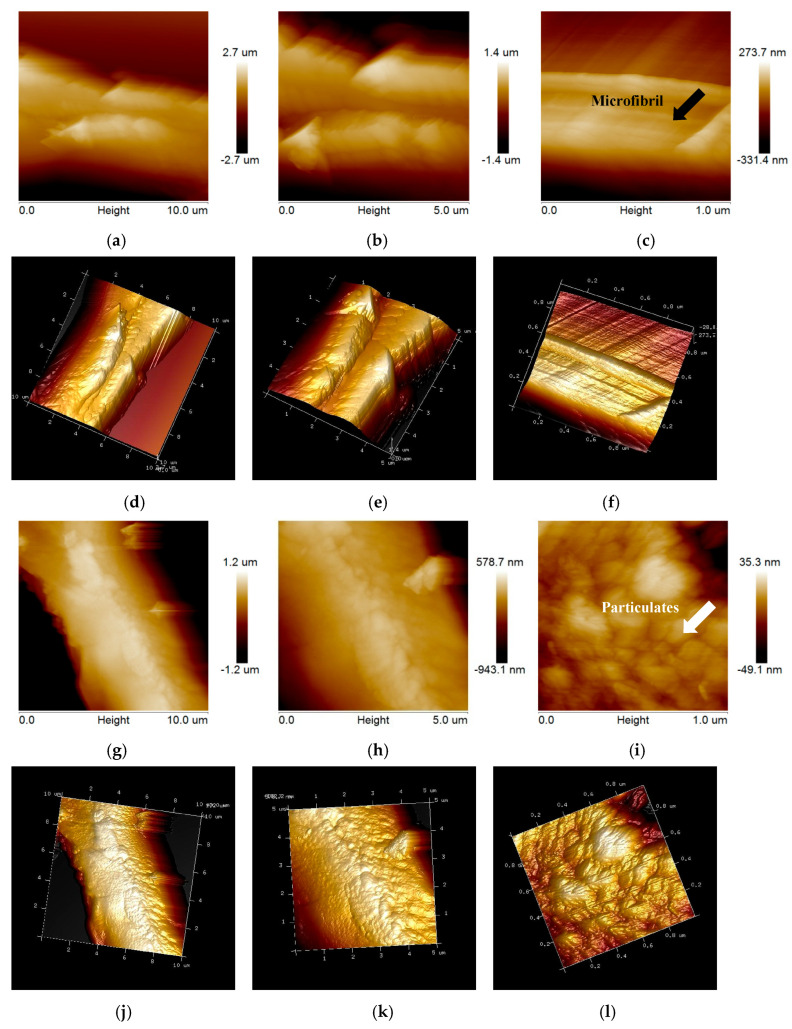
AFM observations of untreated poplar and bisulfite pretreated poplar. In which, (**a**–**c**): phase images of untreated poplar; (**d**–**f**): three-dimensional images of untreated poplar; (**g**–**i**): phase images of bisulfite pretreated poplar; (**j**–**l**): three-dimensional images of bisulfite pretreated poplar.

Figure 4 shows the ultrastructure of untreated and bisulfite pretreated poplar. Different from untreated EFB, some smooth microfibrils were observed in untreated poplar (Figure 4a–f), while particulate matters could be observed in the ultrastructure of untreated EFB (Figure 3c,f). It might be a result of the micro-distribution of chemical compositions of the cell wall polymers (cellulose, hemicelluloses, and lignin) in plant cell walls. After bisulfite pretreatment, great changes were observed in the ultrastructure of poplar cell wall (Figure 4g–l). The orderly microfibril structures were destroyed, and some granular materials were generated, which might be caused by the exposure of hemicellulose, lignin, and extractives during bisulfite pretreatment [32].

Comparing pretreated poplar and pretreated EFB with the same scanning area of 1.0 µm × 1.0 µm (Figure 3i and Figure 4i), the change trends of the structure were different. For non-wood material EFB, large particulate matters turned into a lot of small particulate matters during bisulfite pretreatment, while particulate matters formed for poplar during bisulfite pretreatment, indicating that there were different mechanisms that improved enzymatic hydrolysis efficiency during bisulfite pretreatment of poplar and EFB under the same pretreatment conditions.

It was concluded that the structures of EFB and poplar, including their anatomical structure and ultrastructure, were destroyed, and chemical compositions could be partly removed by bisulfite pretreatment, leading to uncovered cellulose and enhanced cellulase penetration inside the raw materials. Therefore, the enzymatic digestibility of lignocellulose was improved.

### 2.4. Changes in Lignin Distribution of EFB and Poplar Cell Walls by Bisulfite Pretreatment

The distribution of chemical components are also important factors impacting enzymatic degradation. Of them, Lignin has a significant impact on enzymatic hydrolysis efficiency through non-productive adsorption of cellulases and physically hindering enzymes from accessing the cellulose substrate [27]. The application of autofluorescence permits an assessment of the gross localization of lignin in lignified tissues [34]. Autofluorescence with blue light excitation (488 nm) is primarily due to lignin based on its general appearance [35]. Cellulose is also known to be autofluorescent, but the light intensity is generally much dimmer than lignin [36]. CLSM has been developed over the years to analyze the lignin distribution through measuring the relative amounts of lignin in different regions of the cell wall based on the brightness of fluorescence images) [37]. Brighter areas with great fluorescence strength represent high lignin concentration, and increased autofluorescence reflects higher lignin concentration. CLSM is best suited in the visualization of fluorescent structures [38].

**Figure 5:**
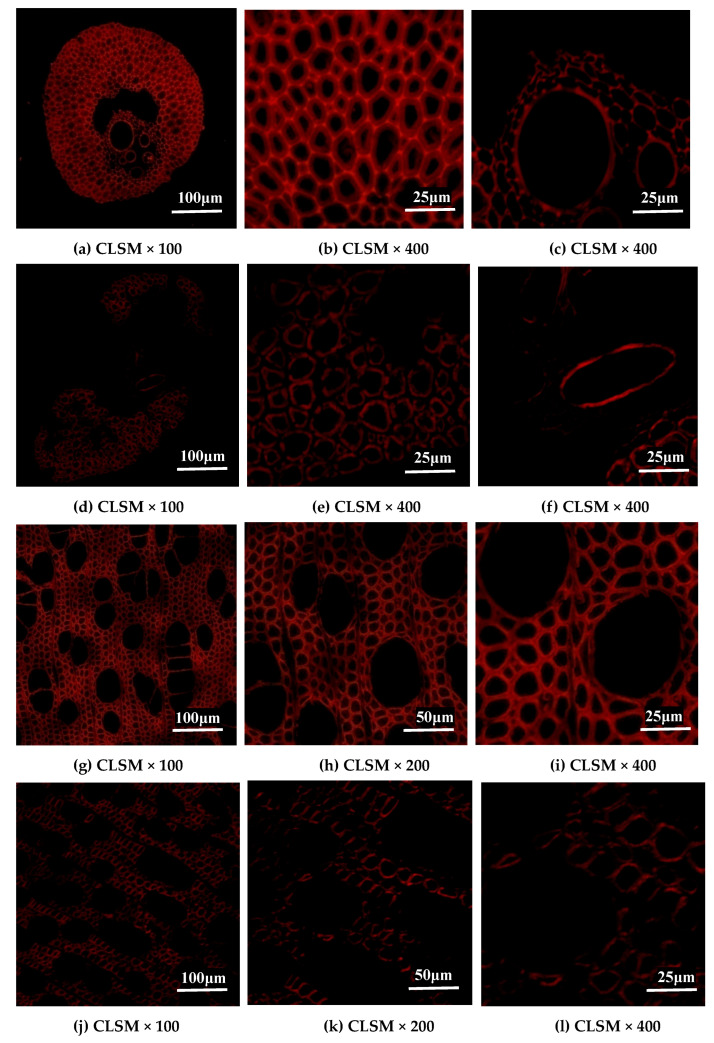
CLSM observations of untreated and bisulfite pretreated EFB and poplar. In which, (**a**–**c**): untreated EFB; (**d**–**f**): bisulfite pretreated EFB; (**g**–**i**): untreated poplar; (**j**–**l**): bisulfite pretreated poplar; in which, (**b**,**e**): sclerenchyma of EFB; (**c**,**f**): parenchyma of EFB.

Figure 5 shows CLSM images of untreated and bisulfite pretreated EFB and poplar samples under different magnifications. Figure 5a–c,g–i indicated the CLSM images of transverse sections of untreated EFB and untreated poplar under different magnifications, respectively. For sclerenchyma of untreated EFB, fluorescence intensities in regions of middle lamella (ML) and cell corner were increased, suggesting strong lignification of ML and cell corner. The fluorescence intensity of secondary wall regions was relatively lower, indicating less lignified secondary walls and higher concentrations of cellulose in there. Meanwhile, CLSM images provided further information, indicating greater autofluorescence of sclerenchyma in contrast to vessels and parenchyma under similar excitation conditions, which was different from other materials presented in the previous studies where vessel walls were more lignified [39,40]. For untreated poplar, observation of xylary fibers with CLSM showed that there was similar strength of autofluorescence in each layer of xylary fibers except for part of ML and primary wall (Figure 5g–i). Vessels and ray parenchyma showed slightly lower levels of autofluorescence compared with xylary fibers of untreated poplar, indicating less lignification of these cells.

Images of bisulfite pretreated EFB (Figure 5d–f) and poplar (Figure 5j–l) were acquired under different magnifications. It was found that bisulfite pretreatment resulted in the great changes in lignin distribution in EFB and poplar. After bisulfite pretreatment, the autofluorescence intensity decreased in all the images of EFB (Figure 5d–f), suggesting that the concentration of lignin in the cell wall was reduced by bisulfite pretreatment. The autofluorescence in the cell corner and ML of the sclerenchyma and parenchyma almost disappeared, indicating that lignin in these regions was largely removed, which made the cell wall separated from each other. The removal of the lignin located in the cell wall corner of sclerenchyma, partial parenchyma, and ML caused the destroyed overall structure of EFB during bisulfite pretreatment, and the sclerenchyma, parenchyma, and vessel in EFB were separated from cell to cell. For bisulfite pretreated poplar, the frame of the poplar was intact after bisulfite pretreatment, while the autofluorescence of the cell wall corner, vessel, and partial ML was less compared with the locations in untreated poplar under the same excitation condition, indicating that the lignin removal mainly occurred in the places of cell wall corner, vessel, and partial ML for poplar during bisulfite pretreatment. The result was consistent with the previous speculation of AFM observation. The removal of lignin in the EFB and poplar and the destroyed overall structure caused by bisulfite pretreatment reduced the adverse effects of lignin on cellulase and expanded the contact area of the cellulosic substrate, thus improving the enzymatic digestibility of EFB and poplar.

## 3. Materials and Methods

### 3.1. Materials

The oil palm EFB originated from Malaysia was used in this study, and the poplar chips were provided by Tianjin University of Science and Technology (Tianjin, China). The EFB and poplar chips were milled to the particle size ranging from 0.30 to 0.45 mm to analyze the chemical composition. To prepare for slicing and bisulfite pretreatment, the EFB and poplar chips were, respectively, cut to the length of 1–1.5 cm and 0.5 × 1 cm.

The cellulase (Sino Enzymes R) with a filter paper activity of 150 IU/g and β-glucosidase activity of 35 IU/g was purchased from Baiyin Sainuo Technology Ltd. (Baiyin, China). Sodium bisulfite, sulfuric acid, glycerol, and ethanol were of analytical grade.

### 3.2. Bisulfite Pretreatment of EFB and Enzymatic Hydrolysis of Pretreated Samples

Bisulfite pretreatment was performed as previously described [20]. Briefly, EFB and poplar chips reacted with a solution of sodium bisulfite (3% NaHSO_3_, 1% H_2_SO_4_) at 180 °C for 30 min. The ratio of solid to liquid in pretreatment was 1:4. After pretreatment, the loss of lignin, xylan, and glucan were calculated according to Formula (1):

Loss rate (%) = (M2 × X)/M1 × 100%(1)

where M1 and M2 represent the percentage contents of lignin, xylan, and glucan before and after pretreatment, respectively (%); X denote the solid yields after pretreatment (%).

The pretreated samples were hydrolyzed in shaking flasks using a commercial cellulase (Sino Enzymes R) to evaluate their enzymatic digestibility. The enzymatic hydrolysis conditions were as follows: 2% solid concentration (on dry substrate weight), pH 4.8 (0.2 M sodium citrate buffer), 45 °C, 150 rpm in a shaker, and cellulase dosage of 20 FPU/g dry substrate.

### 3.3. Assays of Chemical Compositions

Moisture content, ethanol-extractives and different monosaccharide contents were determined according to the analytical procedure of the NREL [41]. Acid-soluble lignin (ASL) and acid-insoluble lignin (AIL) were determined according to Chinese Standard Methods [42].

### 3.4. Determination of Fiber Morphology

In this experiment, samples were disintegrated before testing. The dispersing process was executed according to Chinese Standard Methods [43]. Briefly, samples were dissolved in the mixed solvent of glacial acetic acid and hydrogen peroxide (volume ratio 1:1) at 60 °C for 30–48 h. Then, the dispersed fiber was used to analyze the fiber morphology. The fiber length, width and fines content of samples were determined with fiber quality analyzer (LDAO2, OpTest, Hawkesbury, ON, Canada) before and after bisulfite pretreatment.

All experiments including pretreatment, enzymatic hydrolysis, assays of chemical compositions and morphologic parameters were conducted in triplicates, and the average values were reported in the paper.

### 3.5. Observation of Anatomy and Ultrastructure

The anatomy and ultrastructure of EFB and poplar samples were examined in the present study. Briefly, the untreated EFB and poplar samples were softened, fixed, and embedded with freeze embedding agent (optimal cutting temperature compound, OCT compound). Subsequently, 8-µm-thick transverse sections were prepared with the freezing microtome (MICROM HM 500, Walldorf, Germany).

#### 3.5.1. Light Microscopy (LM) Observation

The 8-µm-thick transverse sections of untreated and pretreated samples were examined using LM (Nikon ECLIPSE E100, Tokyo, Japan) at different magnifications.

#### 3.5.2. Scanning Electron Microscopy (SEM) Observation

A 10 nm-thick gold film was coated on section surfaces of the samples with sputter coater, and then the samples were examined using SEM (JEOL JSM-6700, Tokyo, Japan) at different magnifications.

#### 3.5.3. Atomic Force Microscopy (AFM) Observation

AFM was performed using a Veeco NanoScope IIIA Multimode scanning probe microscope. The images were scanned in the tapping mode in the air using a silicon nitride cantilever probe. The scanning size ranged from 1 to 10 µm, and all the images were measured at a resolution of 512 × 512 pixels. For each sample, many locations were examined but only the representative AFM images were presented here.

### 3.6. Lignin Distribution Analysis

Lignin distribution was characterized using CLSM. The transverse sections of untreated and bisulfite-pretreated EFB and poplar samples were stained with 0.001% (*w*/*w*) acridine orange at room temperature for 2.5 h in the dark. After staining, the sections were carefully washed with deionized water, placed on a glass slide, covered with coverslips and mounted with nail polish. The images of stained sections were acquired using CLSM (Carl Zeiss LSM 700, Waltham, MA, USA). An argon laser at λ = 488 nm was used as the excitation light source, and fluorescence emission (λ = 590 nm) was collected as the lignin [43].

## 4. Conclusions

It was demonstrated that bisulfite pretreatment significantly enhances the enzymatic digestibility of EFB and poplar, and the glucose yields increased by about 3.85 times for EFB and 2.54 times for poplar, respectively, after pretreatment and enzymatic hydrolysis compared to untreated samples. The effects of bisulfite pretreatment on the anatomical structure and ultrastructure of the cell wall, and the distribution of lignin in the cell wall of EFB and poplar were investigated to explore the improvement mechanisms of enzymatic digestibility by bisulfite pretreatment. It was shown that bisulfite pretreatment can remove a significant portion of xylan (79.4% and 87.3%, respectively) and part lignin (39.0% and 33.8%, respectively) from EFB and poplar, which destroyed the anatomical structure and ultrastructure of the cell wall, and changed the distribution of lignin in the cell wall. Lignin removal primarily occurs in the cell wall corners of sclerenchyma, partial parenchyma, and middle lamella of EFB, as well as in the cell wall corners, vessels, and partial middle lamella of poplar. These changes in chemical compositions and structures increase the accessibility of enzymes to cellulose and reduce the adverse effects of lignin on cellulase such as the steric hindrance and nonproductive adsorption of lignin, thus enhancing enzymatic digestibility. The study also reveals the differences in the structural characteristics of non-wood EFB and wood poplar and their changes before and after bisulfite pretreatment.

## Figures and Tables

**Table 1:** The changes in chemical compositions and enzymatic digestibility of EFB and poplar before and after bisulfite pretreatment ^a^.

	Yield (%)	Extractives (%)	Glucan (%)	Xylan (%)	Lignin (%)	Lignin (%) (% loss)	Xylan (%) (% loss)	Glucan (%) (% loss)	Glucose Yield ^b^ (g/g)
Untreated EFB	—	1.5 ± 0.3	34.7 ± 1.1	23.1 ± 1.3	22.1 ± 1.2	—	—	—	0.078
Pretreated EFB	65.8 ± 3.9	7.6 ± 1.9	51.8 ± 3.5	7.2 ± 2.1	20.5 ± 3.2	39.0	79.4	1.8	0.378
Untreated Poplar	—	1.3 ± 0.1	43.7 ± 1.7	14.6 ± 1.0	23.1 ± 0.9	—	—	—	0.088
Pretreated Poplar	71.7 ± 4.1	10.6 ± 1.8	58.5 ± 3.2	2.6 ± 1.3	21.3 ± 3.9	33.8	87.3	4.0	0.312

^a^ Bisulfite pretreatment conditions: 1% H_2_SO_4_, 3% NaHSO_3_, 180 °C, 30 min. ^b^ Glucose yield at 72 h of enzymatic hydrolysis based on the oven-dried weight of untreated EFB or untreated poplar respectively.

**Table 2:** Fiber morphology parameters of EFB and poplar before and after bisulfite pretreatment.

	L (n) ^a^ (mm)	L (w) ^b^ (mm)	Width (µm)	Fine (%)
Untreated EFB	0.525 ± 0.001	0.674 ± 0.001	17.234 ± 0.036	24.35 ± 0.226
Pretreated EFB	0.425 ± 0.001	0.530 ± 0.006	16.283 ± 0.138	41.22 ± 0.184
Untreated Poplar	0.632 ± 0.014	0.721 ± 0.015	20.408 ± 0.654	14.89 ± 0.219
Pretreated Poplar	0.397 ± 0.001	0.482 ± 0.001	17.870 ± 0.099	60.12 ± 2.192

^a^ Number average of fiber length. ^b^ Weight average of fiber length.

## Data Availability

Data are contained within the article.
